# Target Development towards First Production of High-Molar- Activity ^44g^Sc and ^47^Sc by Mass Separation at CERN-MEDICIS

**DOI:** 10.3390/ph17030390

**Published:** 2024-03-18

**Authors:** Edgars Mamis, Charlotte Duchemin, Valentina Berlin, Cyril Bernerd, Mathieu Bovigny, Eric Chevallay, Bernard Crepieux, Vadim Maratovich Gadelshin, Reinhard Heinke, Ronaldo Mendez Hernandez, Jake David Johnson, Patrīcija Kalniņa, Alexandros Koliatos, Laura Lambert, Ralf Erik Rossel, Sebastian Rothe, Julien Thiboud, Felix Weber, Klaus Wendt, Rudolfs Jānis Zabolockis, Elīna Pajuste, Thierry Stora

**Affiliations:** 1 European Organization for Nuclear Research (CERN), Esplanade des Particules 1, 1211 Geneva, Switzerland; 2 Institute of Chemical Physics (ICP), University of Latvia, Jelgavas Street 1, LV-1004 Riga, Latvia; 3 Institut für Physik, Johannes Gutenberg Universität, Staudingerweg 7, 55128 Mainz, Germany; 4 Instituto Superior de Tecnologías y Ciencias Aplicadas, Universidad de La Habana (InSTEC-UH), Ave. Salvador Allende No. 1110 e, Infanta y Rancho Boyeros, Plaza de la Revolucion, La Habana 10400, Cuba; 5 Department of Physics and Astronomy (IKS), Katholieke Universiteit Leuven, Celestijnenlaan 200D, 3010 Heverlee, Belgium

**Keywords:** scandium radionuclides, mass separation, ISOL target units, target materials, laser resonant ionization, molecular beams

## Abstract

The radionuclides ^43^Sc, ${\;\;}^{44g/m}$Sc, and ^47^Sc can be produced cost-effectively in sufficient yield for medical research and applications by irradiating ${\;\;}^{nat}$Ti and ${\;\;}^{nat}$V target materials with protons. Maximizing the production yield of the therapeutic ^47^Sc in the highest cross section energy range of 24–70 MeV results in the co-production of long-lived, high-γ-ray-energy ^46^Sc and ^48^Sc contaminants if one does not use enriched target materials. Mass separation can be used to obtain high molar activity and isotopically pure Sc radionuclides from natural target materials; however, suitable operational conditions to obtain relevant activity released from irradiated ${\;\;}^{nat}$Ti and ${\;\;}^{nat}$V have not yet been established at CERN-MEDICIS and ISOLDE. The objective of this work was to develop target units for the production, release, and purification of Sc radionuclides by mass separation as well as to investigate target materials for the mass separation that are compatible with high-yield Sc radionuclide production in the 9–70 MeV proton energy range. In this study, the in-target production yield obtained at MEDICIS with 1.4 GeV protons is compared with the production yield that can be reached with commercially available cyclotrons. The thick-target materials were irradiated at MEDICIS and comprised of metallic ${\;\;}^{nat}$Ti, ${\;\;}^{nat}$V metallic foils, and ${\;\;}^{nat}$TiC pellets. The produced radionuclides were subsequently released, ionized, and extracted from various target and ion source units and mass separated. Mono-atomic Sc laser and molecule ionization with forced-electron-beam-induced arc-discharge ion sources were investigated. Sc radionuclide production in thick ${\;\;}^{nat}$Ti and ${\;\;}^{nat}$V targets at MEDICIS is equivalent to low- to medium-energy cyclotron-irradiated targets at medically relevant yields, furthermore benefiting from the mass separation possibility. A two-step laser resonance ionization scheme was used to obtain mono-atomic Sc ion beams. Sc radionuclide release from irradiated target units most effectively could be promoted by volatile scandium fluoride formation. Thus, isotopically pure ${\;\;}^{44g/m}$Sc, ^46^Sc, and ^47^Sc were obtained as mono-atomic and molecular ScF${\;\;}_{2}^{+}$ ion beams and collected for the first time at CERN-MEDICIS. Among all the investigated target materials, ${\;\;}^{nat}$TiC is the most suitable target material for Sc mass separation as molecular halide beams, due to high possible operating temperatures and sustained release.

## 1. Introduction

Theranostics is a rapidly evolving personalized nuclear medicine approach, combining therapy and diagnostics. It offers cellular-cancer diagnostics followed by non-invasive tumor treatment using drugs labeled with radionuclides. Most commonly, such theranostic drug pairs are created with radionuclides of different chemical elements, such as diagnostic ^68^Ga or ^18^F paired with therapeutic ^177^Lu [1]. Therefore, the drug kinetics and sometimes their exact physico-chemical properties do not exactly match when therapeutic and diagnostic agents are compared. To resolve this issue, radioisotopes of the same chemical element can be used to create so-called “matched pair” radiopharmaceuticals. They have the same chemical properties and pharmacokinetics that help in the efficient planning and monitoring of personalized targeted cancer treatment [2]. With the use of “matched pair” radiopharmaceuticals, the received-dose estimation and pharmacokinetics in the body could better be understood and, therefore, optimized [3]. A perfect candidate for “matched pair” radiopharmaceuticals development is scandium with ${\;\;}^{43/44g}$Sc, both applicable in Positron-Emission Tomography (PET) and ^47^Sc (T${\;\;}_{1/2}$ = 3.35 d) as the therapeutic match with a γ-ray emission (E${\;\;}_{\gamma }$ = 159.4 keV (68.3%)) suitable for Single-Photon-Emission Computed Tomography (SPECT). Another advantage is that these Sc radionuclides decay into bio-compatible chemical elements, namely Ca and Ti. While ^43^Sc (T${\;\;}_{1/2}$ = 3.89 h) and ${\;\;}^{44g}$Sc (T${\;\;}_{1/2}$ = 4.04 h) have similar half-life and positron emission probabilities, ^43^Sc shows an advantage for the imaging technique in comparison with ${\;\;}^{44g}$Sc by the absence of high-energy γ-ray emission (1157 keV) [4].

The availability of ${\;\;}^{43,44g}$Sc and ^47^Sc for clinical studies is still very limited, and their production is mostly undertaken with enriched titanium and calcium target materials. The most common and efficient production of ^43^Sc and ${\;\;}^{44g}$Sc proceeds via cyclotrons using low-energy protons (<30 MeV), deuterons, or alpha particles on natural and enriched calcium targets. The ^43^Ca, ^44^Ca, and ^46^Ca isotopes are naturally present in low quantities in ${\;\;}^{nat}$Ca. However, due to limited enrichment and abundance, the use of enriched Ca targets significantly increases production costs. Furthermore, ${\;\;}^{44m}$Sc (T${\;\;}_{1/2}$ = 58.61 h) is co-produced in most cases (see Table 1) [5]. The ^47^Sc radionuclide, as the therapeutic counterpart in an Sc “matched pair”, can be efficiently produced in nuclear reactors by activating ^47^Ti, which is naturally present in ${\;\;}^{nat}$Ti (^46^Ti 8.25%, ^47^Ti 7.44%, ^48^Ti 73.72%, ^49^Ti 5.41%, ^50^Ti 5.18%) or by thermal neutron capture on ^46^Ca resulting in ^47^Ca/^47^Sc generator [6,7]. The scarce availability of nuclear reactors with the necessary neutron energy has fostered accelerator use for the production. The most common accessible and commercially available cyclotrons can accelerate protons and in some cases deuterons and alpha particles to energies up to 18 MeV. Several routes—by using intermediate-(<30 MeV)-energy or medium-(30–70 MeV)-energy cyclotrons and enriched-titanium, -calcium, or -vanadium target materials and accelerated protons or deuterons—have been investigated for the production of ^47^Sc. All of them present similar production cross sections (ranging from 30 to 60 mb) but show different production costs, due to the availability of the enriched material [8]. Less common production routes involve photonuclear reactions on enriched ^48^Ti target material [9] as well as ${\;\;}^{44}$Ca and ${\;\;}^{51}$V irradiation with alpha beams [10]. As in ${\;\;}^{44g}$Sc production, ${\;\;}^{47}$Sc is accompanied by long-lived, high-γ-ray-energy ${\;\;}^{46}$Sc and ${\;\;}^{48}$Sc contaminants (see Table 1).

**Figure 1:**
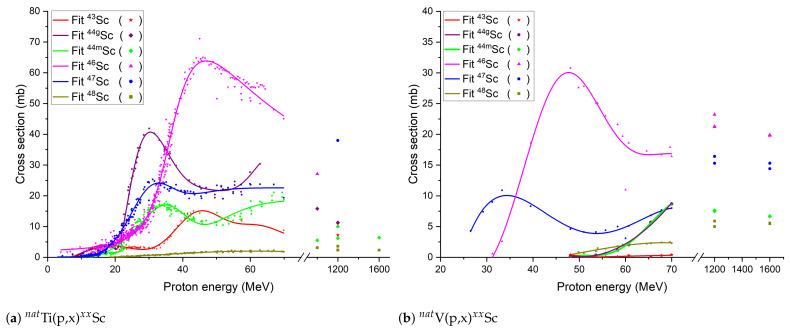
Fit of ${\;\;}^{nat}$Ti and ${\;\;}^{nat}$V excitation experimental data for Sc radionuclide production with protons up to 1.6 GeV [13].

Although acceptable radionuclidic-purity Sc radionuclide production with cyclotrons and nuclear reactors is possible from enriched target materials (e.g., ${\;\;}^{43,44,46,48}$Ca), this route is not sustainable in terms of production cost or availability to neutron irradiation. An alternative, more cost-efficient way would be the use of natural titanium or vanadium target materials. Utilizing ${\;\;}^{nat}$V as a target for ${\;\;}^{47}$Sc production requires the use of medium-energy protons and leads to a quite-low production yield, due to a low cross section (<11 mb). If one wants to avoid the production of ${\;\;}^{46}$Sc in the final product, the cross section falls to 7.4 mb [19]. Compared to ${\;\;}^{nat}$V, ${\;\;}^{nat}$Ti irradiation by protons with cyclotrons yields higher Sc radionuclide production; however, a mix of the same long-lived, high-γ-ray ${\;\;}^{46}$Sc and ${\;\;}^{48}$Sc contaminants are produced (see Figure 1). Therefore, the irradiated targets must go through a mass separation step to remove the ${\;\;}^{46}$Sc and ${\;\;}^{48}$Sc contaminants before or after any radiochemical separation. As indicated in Table 1, mass separation is required to separate ${\;\;}^{43}$Sc from ${\;\;}^{44m}$Sc, as well as for purification of ${\;\;}^{44g/m}$Sc and ${\;\;}^{47}$Sc that is not possible with any other conventional methods or even enriched target materials.

Electromagnetic-ion-beam mass separation is a technique that uses a dipole magnet to induce a magnetic field and separate accelerated ions according to their mass-to-charge ratio, through the Lorentzian principle. The magnetic rigidity of the ion beam of interest depends on the applied magnetic field from the dipole as well as the charged state of the ion, mass, and energy. By blocking out unwanted trajectories, only pure beams of interest can be selected. In contrast to chemical separation, isobars or atoms with the same mass number (A) but different atomic numbers (Z) are separated from the initial sample. The quality of a mass separator is expressed by the mass resolving power, and certain mass separators have sufficient resolution to separate isobars [20,21]. CERN-ISOLDE (Isotope Separation On-Line DEvice) in Switzerland is a facility that has utilized this technique for decades to deliver isotopes for atomic, nuclear, and solid-state physics experiments [22,23]. The mass separation technique was recently adapted by the CERN-MEDICIS (MEdical Isotopes Collected from ISolde) offline mass separator to obtain isotopically pure non-conventional medical radionuclides. These mass separated radionuclides are implanted into thin metallic or salt-coated foils and dispatched to biomedical or radiochemical research centers worldwide [24]. To test target units, study target materials, ion sources, and stable (non-radioactive) isotopes and molecules, two offline mass separators (Offline-1 and Offline-2) are available [25].

Sc radionuclides have been produced at ISOLDE before; however, the conditions of their efficient release were extreme, not optimized, and not suitable for lower-melting-point-(<2000 °C) target materials. Spallation reactions of ${\;\;}^{nat}$Ta foils are the primary source in obtaining Sc isotopes at ISOLDE for physics experiments, due to the stability of Ta at very high operating temperatures (>2000 °C). However, the production yield of medically relevant Sc radionuclides with mass separation from ${\;\;}^{nat}$Ta is not yet applicable for medical research [26]. Furthermore, ${\;\;}^{nat}$Ta spallation requires high-energy protons that are not available in typical intermediate- or medium-energy cyclotron centers.

Refractory-element release from the target units (e.g., Si, B, C, Sc, Ti, V, and transition metals Nb, Mo, Tc, W, Re, Os) can be promoted with in situ chemical reactions in the target containers and ion sources by creating more volatile molecules and can be extracted from target units as molecular-ion species [27]. Molecular-beam formation is achieved by either injecting reactive gas, such as CF${\;\;}_{4}$ and SF${\;\;}_{6}$ [28], CO [29], or evaporating salts (e.g., CCl${\;\;}_{4}$, AgCl, BF${\;\;}_{3}$) from a separate container within the target unit. Molecular-beam formation can also be observed from impurities in raw target materials or from its chemical composition (e.g., LaF${\;\;}_{3}$, Ce${\;\;}_{3}$S${\;\;}_{4}$, CeS) [30,31]; however, in such cases, the reaction rate cannot be controlled during operations.

This study was motivated by the potential and benefits of combining the novel mass separation method with conventional cyclotron-compatible, cost-efficient target materials to obtain high molar activity and isotopically pure Sc radionuclides. The objective of this work was to investigate, test, and determine the most suitable target and ion source units as well as cost-efficient target materials for medical Sc radionuclide production and mass separation at CERN-MEDICIS. During this study, natural vanadium foils were used for the first time for radioactive ion beam production at CERN. Various halogenating (NF_3_, Cl_2_, CF_4_) gases as well as target units, ion sources, and operational parameters were systematically tested with these target materials for Sc molecular ion beam formation and extraction. In this study, a new two-step laser resonance mono-atomic Sc ionization scheme was tested and used to obtain Sc ion beams from irradiated target materials. Also, the first molecular ScF${\;\;}_{x}^{+}$ (X=1−2) beams at MEDICIS and the first ${\;\;}^{44g/m}$ScF${\;\;}_{2}^{+}$, ${\;\;}^{45}$ScF${\;\;}_{2}^{+}$, ${\;\;}^{46}$ScF${\;\;}_{2}^{+}$, and ${\;\;}^{47}$ScF${\;\;}_{2}^{+}$ radioactive molecular beams were observed and used for Sc isotope mass separation. Each investigation in this study is reflected in a separate section showing the context and historical data and explaining the applicable methods and equipment, followed by the observed findings and results. This way, the potential of performing mass separation of ${\;\;}^{43}$Sc, ${\;\;}^{44g/m}$Sc, and ${\;\;}^{47}$Sc from naturally abundant, irradiated thick MEDICIS targets as well as externally irradiated samples to obtain high molar activity and radiochemically pure Sc radionuclides is systematically shown.

## 2. Thick Targets and Mass Separator

### 2.1. Historical Target Materials at ISOLDE

The most common thick-target materials used at ISOLDE for radioactive ion beam production are uranium carbide (UC${\;\;}_{x}$) or metallic natural tantalum (${\;\;}^{nat}$Ta) foils [32,33], which are also used at MEDICIS for radiolanthanide production and mass separation [34]. Various forms of ${\;\;}^{nat}$Ti and ${\;\;}^{nat}$V target materials capable of producing Sc isotope beams have been used at ISOLDE in the past 40 years [32] and are summarized in Table 2. TiO${\;\;}_{2}$, Ti${\;\;}_{2}$O${\;\;}_{3}$, Ti${\;\;}_{5}$Si${\;\;}_{3}$, and TiN target materials for isotope separation on-line (ISOL) have been investigated by Oak Ridge National Laboratory (US) and TRIUMF (Canada), but only for ${\;\;}^{48}$V release [35]. Ti, VC, and TiC target materials have been fluorinated with CF${\;\;}_{4}$ gas addition during operations at ISOLDE to promote Sc release, but the extraction of mono-atomic Sc even at 1900 °C from target units was slow and did not improve with the CF${\;\;}_{4}$ addition [28,35] or was obtained in conditions where the target material was molten and being evaporated [36].

Because of the insufficient yield (atoms per μC incident proton beam), slow isotope release from the target unit, melting point of the material, powder sintering, or element selectivity, these materials were discarded from routine use. On the other hand, such a controlled and sustained release approach would be suitable for collections at MEDICIS for radionuclides, where single-batch collections can last for days.

Refractory Sc release from the ISOLDE and MEDICIS target units has proven to be very challenging, due to the high boiling point, low vapor pressure, and high chemical reactivity of scandium. The refractory nature of Sc is one of the reasons for the low yields as well as the inconsistent beam-delivery results obtained for mass separation up to now. Titanium has higher vapor pressure and lower melting temperature than vanadium [35]; however, only V powder has been used as target material at ISOLDE. Powders tend to sinter more easily and at lower temperatures, thereby lowering the extracted isotope yield over time [20]. Before MEDICIS, there was limited interest in sustained slow mono-atomic or molecular-scandium-isotope ion beam release and production of medically suitable activities, which explains why no further investigations of these target materials were done.

### 2.2. Mass Separator

**Figure 2:**
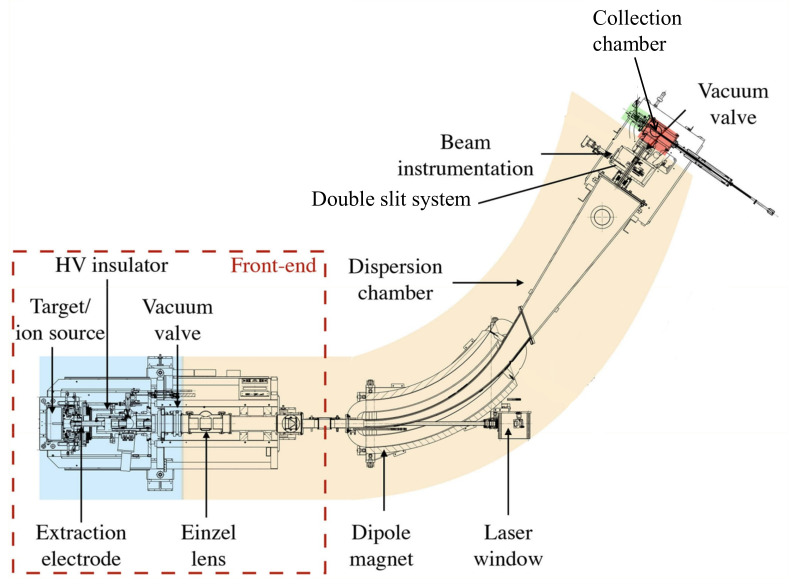
MEDICIS mass separator beamline schematics. Adapted from [37].

In this study, two mass separators were used to develop and extract the scandium ion beams. Radioactive Sc ion beams were obtained and mass separated by the MEDICIS offline mass separator. The MEDICIS mass separator schematic is shown in Figure 2. The first part is a front-end for the radioactive ion beam production, acceleration, and optics. The front-end consists of:

- an electrically isolated target-unit coupling system, operated at 30–60 kV potential;
- a target and ion source unit that is exchanged for each production batch;
- a Ti extraction electrode that is placed at 50–80 mm distance from the ion source;
- an Einzel lens to shape the ion beam, operated at 11–26 kV, depending on the extraction potential and ion source type;
- horizontal and vertical electrostatic deflectors (±200–600 V).

The MEDICIS mass separator section consists of a 55° double-focusing magnet with a bending radius of 1.5 m and is operated up to 90 A current [37]. A dedicated window opposite the front-end is used to reflect the laser beam directly into the ion source. Before the bending magnet, a copper Faraday cup is inserted into the beam path to measure the total beam current entering the dipole magnet. The beam instrumentation box after the magnet houses a wire scanner to assess the beam shape and position, another Faraday cup to measure the separated beam current, and a recently upgraded double-slit system, which allows for isolating single or two-ion different-mass ion beams simultaneously, with a mass difference of 1–3 amu [34]. Last on the beamline is the sample collection chamber, where the sample holder with up to three collection foils (15 × 20 mm) is placed in the radioactive ion beam path to implant the isotope(s) of interest. The sample holder consists of a collimator, an electron repeller, and a sample plate into which the impinging beam current can also be monitored. The produced ions are collected on Zn- or Al-coated gold foils, full Al foils, or salt-covered Al foils in a position perpendicular or angular to the ion beam.

The MEDICIS mass separator is operated at a pressure of 1 $\times \;10^{-5}$–1 $\times \;10^{-7}$ mbar. Neutral radioactive isotopes are pumped from the separator volume and are stored in local tanks until their decay to a sufficiently low level for release into the atmosphere [37]. Every separator section can be isolated by valves. Reactive and noble gas injection into the target units is done through a dedicated gas supply system that is located inside the high-voltage-and-power-supply room, outside the separator bunker. Another isolated gas system is connected to the same feed line to the target for Cl${\;\;}_{2}$ injection and residual-gas neutralization. Up to 1.4 bar pressure can be applied to the target-unit gas leaks.

Short γ spectroscopy measurements during radioactive ion beam collection are performed online with a 1 cm cubed Cadmium Zinc Telluride (CZT) γ-ray Kromek GR1+ semiconductor detector that is placed directly behind the sample foil [38]. This way, the radionuclide activity that is collected on the collection foils can be monitored during the whole experiment. Two minutes of real-time acquisitions were done every 5 min. The resolution of GR1+ is <2.0% FWHM @ 662 keV [39]. The obtained spectra were acquired, saved, displayed, and quantitatively analyzed by the Kromek Multi-Spect Analysis spectroscopy software (Version 14.24.3.243). In addition, high resolution γ-spectroscopy measurements were performed on every retrieved sample after collection and radiochemistry procedure. The spectra were taken with an HPGe coaxial detector from MIRION Technologies (Canberra) S.A.S. The energy range of the detector is 3–10,000 keV, with relative efficiency of >40% and resolution of <1.2 keV (at 122 keV) and <2.0 keV (at 1332 keV) [40]. These latter measurements were used to quantify the separation efficiency at the end of the collections and any possible sputtering effect [41].

Before proceeding to the extraction of radioactive Sc isotopes at MEDICIS, an offline mass separator (ISOLDE Offline-1) was used for target-unit testing before irradiation and for stable (non-radioactive) beam developments. The layout of the Offline-1 separator is similar to the MEDICIS one; however, the target unit is operated only at 30 kV [21]. Another recent Offline-1 upgrade includes a collection chamber at the end of the instrumentation box, where a stable ion beam can be implanted in metallic foil, released by resistively heating the foil, and detected by a residual gas analyzer (1–200 amu) at the top of the collection chamber [25]. A separate target coupling table (pump stand) having the possibility to apply current for heating and to reach a vacuum inside the target units down to 1 $\times \;10^{-7}$ mbar was used to calibrate each target container and transfer line temperature according to the applied current. It was also used for outgassing of the ${\;\;}^{nat}$TiC pellet charge.

## 3. Scandium Radionuclide Production Yields

### 3.1. Radionuclide Production at MEDICIS

In this study, the target materials were irradiated at CERN-MEDICIS with a 1.4 GeV proton beam delivered by the Proton Synchrotron Booster (PSB). The target units were placed downstream of an ISOLDE target unit. An increase of proton energy to 2 GeV is planned in a few years. The proton beam first travels through an empty ISOLDE aluminium target-unit vessel (direct beam) or a full target container (indirect beam). Therefore, the irradiation parameters at CERN-MEDICIS and the conditions strongly depend on an ongoing ISOLDE experiment (see [24] for more details). The pulsed proton beam impinges on the target units with up to 2.0 μA intensity. The PSB cycle consists of up to 3.3 $\times \;10^{13}$ protons per 2.4 μA pulse every 1.2 s. In addition to the two different modes of irradiation (direct and indirect), there are two irradiation stations available. The irradiation stations are named after the ISOLDE separators: namely, the General-Purpose Separator (GPS) and the High-Resolution Separator (HRS) [42]. MEDICIS also utilizes another mode of operation, thanks to the institutes that are part of the MEDICIS collaboration. The target materials irradiated at external cyclotron or reactor facilities can be shipped to MEDICIS and placed inside an empty target-unit container and the radionuclide of interest subsequently mass separated [24].

MEDICIS operates in a so-called “batch mode” of production and extraction, meaning that the starting activity of the radionuclide within the inventory for each experiment is a finite amount and decreases from both the radioactive decay and release of the nuclides throughout the collection. This is the main difference between an offline and an online isotope mass separator facility such as ISOLDE. ISOLDE typically operates with a proton beam constantly impinging on the target during mass separation. Therefore, the efficiency in the context of an offline mass separator is defined as the decay-corrected amount (activity) of radionuclides collected at the end of the collection against the amount at the start of the collection. The target materials are irradiated at room temperature and in an Ar atmosphere. No active target cooling is provided. Most of the irradiated target units used for radionuclide release and collections at MEDICIS are re-irradiated and re-used; therefore, multiple collection batches are performed from the same target unit. The irradiation times usually range from a few hours up to a full day (24 h). Every target unit for irradiation in the ISOLDE target area is transported via an automatic rail conveyor system. After irradiation, the dose rate at 27 cm of the retrieved target unit is measured at the MEDICIS decay station. Thus far, the target-unit dose rate has reached 3 Sv/h, 15 min after the End Of the Beam (EOB). It prevents any sample fraction removal for spectroscopy analysis. The target units are transported and placed on the MEDICIS front-end by remote handling, using a KUKA${\;\;}^{®}$ (Augsburg, Germany) robot [24]. Due to the high dose rate and amount of radionuclides produced inside each target, spectrometry of the whole target unit is also not yet feasible.

The radionuclide inventory from each production is predicted via the use of the FLUKA (version 4.3) multi-purpose Monte Carlo code. It covers nucleus–nucleus, hadron–nucleus, and hadron–hadron interactions from their threshold up to 10 PeV energy. FLUKA includes multiple models to describe the interactions. For the energy of 1.4 GeV that concerns our study, the PreEquilibrium Approach to NUclear Thermalization (PEANUT) model is used [21,43,44]. The FLUKA code has been benchmarked with experimental data from radionuclide production in the corresponding energy range [45,46,47]. Flair (version 4-3.1), an advanced interface for several Monte Carlo codes originally developed for FLUKA, was used for visualization and modeling of the geometry as well as processing the results generated from the FLUKA input [48].

ActiWiz (version 3.5.9/2021-2907), another computational tool, was used in this study. While it allows for calculating the production of each isotope from a set of particle fluence spectra as a function of the particle type and energy [49,50], it can also import nuclide production rates from external general-purpose Monte Carlo codes and derive the nuclide inventories by solving the Bateman equation. In addition, it includes many post-processing options to directly analyze the results, which is what the tool was used for in this context.

### 3.2. Theoretical In-Target Production and Thick-Target Yield Results

**Figure 3:**
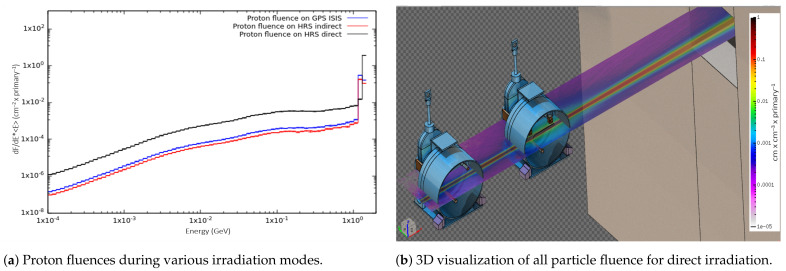
Particle fluences for MEDICIS ${\;\;}^{nat}$Ti foil target charge irradiation with 1.4 GeV protons.

After irradiation of the target units for Sc release and mass separation, γ-spectroscopy measurements were impossible, due to the dose rate of the targets (0.3–1.0 Sv/h at 27 cm distance). The radionuclide inventory for yield estimation was computed with FLUKA and ActiWiz. To estimate the production yield, a few approximations and assumptions had to be considered:

- The target-material nucleus inside the particle-tracking (scoring) region in FLUKA was uniformly distributed. The same was assumed for more complex chemical compounds and molecules, as FLUKA does not take into account the chemical structure of the compound;
- The metallic foil rolls were loaded directly inside the target container and, therefore, the loaded charge density was uniformly distributed and less than the corresponding pure material density. Depending on the weight and foil layers, the obtained charge densities in this study varied from 0.5–1.5 g/cm${\;\;}^{3}$. Even though the actual prepared ${\;\;}^{nat}$V-foil target charge densities resulted in <1 g/cm${\;\;}^{3}$, the highest prepared-metallic-foil charge density was 1.5 g/cm${\;\;}^{3}$ (for ${\;\;}^{nat}$Ti) and was, therefore, taken as a reference for ${\;\;}^{nat}$V in-target production estimations as well;
- The target-material geometry was not precisely defined in the code, and the nucleus distribution assumption was close enough, due to the particle fluence distribution and small energy loss in the target container region (see Figure 3a,b);
- The ${\;\;}^{nat}$TiC pellet charge was confined in a carbon sleeve; therefore, the radionuclide production region in this case was the inner-carbon-sleeve volume that was taken up by the pellets. The rest of the space inside the target container was taken up by the carbon sleeve or left empty. For this reason, the mass of all out-gassed pellets per their initial volume was assumed, resulting in 2.7 g/cm${\;\;}^{3}$ density.

FLUKA production predictions give a good relative comparison of the proposed materials to maximize on direct Sc radionuclide or ${\;\;}^{44}$Ti/${\;\;}^{44g}$Sc and ${\;\;}^{47}$Ca/${\;\;}^{47}$Sc generator production feasibility. One should note the co-production of light radioactive N, C, B, Be, and Li species from carbon in the sleeve and ${\;\;}^{nat}$TiC; however, only ${\;\;}^{7}$Be and ${\;\;}^{10}$Be are long-lived and the rest are very short-lived isotopes [13,51]. Table 3 shows a comparison of production yields with statistical uncertainties for target materials used in this study at various irradiation stations and modes. The yields were simulated with 1 $\times \;10^{6}$ primary particles (protons) and the results are given as produced nuclides per gram of user-defined target material per primary particle. For indirect irradiation, a target unit with a depleted uranium carbide target charge was considered upstream of the proton beam, because it is the most widely used target for radioactive ion beam productions at ISOLDE [33].

Table 4 provides a comparison of the Thick-Target production Yields (TTYs) that can be achieved at MEDICIS and with cyclotrons. The yield of Sc radionuclides and proton fluence per primary particle at MEDICIS is 11 times higher in the case of direct irradiation in comparison with indirect irradiation on the HRS station. Also, the yield from direct irradiation on HRS is 7 times higher than GPS indirect irradiation (ISIS table). The yields from indirect irradiation on GPS are higher than HRS, due to less proton beam straggling because of the difference in distance between the ISOLDE and MEDICIS targets on HRS (530 mm) and GPS (240 mm).

Based on FLUKA computations, Thick-Target Yields (TTYs) were estimated with 1 h irradiation time and no cooling time after the End of the Beam (EOB). The obtained results are summarized in Table 4. To estimate the highest production yield that can be achieved with charge densities for Ti and V foils of 1.5 g/cm${\;\;}^{3}$ and 2.7 g/cm${\;\;}^{3}$ for ${\;\;}^{nat}$TiC, full target charges were assumed. Full-target-charge mass of 100 g for double-foil ${\;\;}^{nat}$Ti, double-foil ${\;\;}^{nat}$V, and 65 g (154 pellets) for ${\;\;}^{nat}$TiC were considered. Based on the particle fluence in Figure 3, the production yield was calculated for direct irradiation on the HRS station; hence, the highest production yield.

Production of TTYs from metallic foils and pressed TiC targets with cyclotrons were estimated, considering a realistic scenario of targets with 1 mm thickness. For comparison, no cooling time after the EOB was considered. Energies of 30 and 70 MeV proton beams were considered. The energy degradation inside the targets for the TTYs calculation was simulated with the SRIM software [52]. The average value between impinging-beam and residual-beam energy was used to obtain an estimate of the yield. To obtain the cross sections at the desired proton energy range, a fit from experimental data, available from the EXFOR database in Figure 1, was performed. The TENDL-2023 [53] theoretical cross section database was used for radionuclides that lack experimental data. Obtained TTYs for MEDICIS targets are relatively higher due to target thickness; however, the MEDICIS target materials are irradiated with a 2 μA proton beam whereas cyclotrons can reach up to 100–350 μA intensities, eventually placing both irradiation modes in the same yield range. One should note that to estimate achievable activity per production run, the possibility of increasing the cyclotron target thickness as well as the mass separation efficiency (0.1–50%, depending on the chemical element) should be taken into account [34,54].

## 4. Target and Ion Source Developments

### 4.1. Target and Ion Source Units

**Figure 4:**
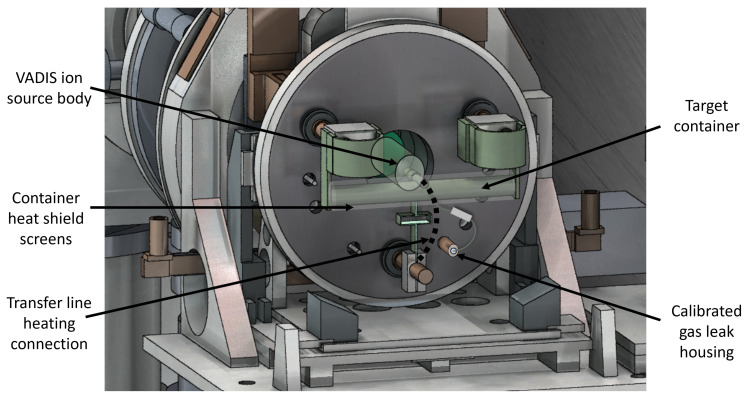
VADIS VD-5 target unit schematic on mass separator front-end without aluminium vessel.

The target and ion source units that are used for radioactive ion beam production and mass separation at MEDICIS generally are identical to the ones being developed for and used at ISOLDE [21,37]. The target unit typically consists of three main parts: an aluminium base and enclosure, a target container and its accessories, and an ion source. Each target unit produced for either MEDICIS or ISOLDE can differ in its specification, modification, and ion source to maximize longevity, production yield, and ionization of the species of interest. Since 1990, more than 840 target units in total have been produced, and some of them are re-used multiple times until dismantling and long-term storage [55]. As shown in Figure 4, the target container and ion source are fixed to the target base, which is water-cooled to temperatures <20 °C during operation. Finally, the target container and ion source assembly with their accessories are enclosed by an aluminium vessel to obtain a pressure below $\times 10^{-5}$ mbar. The target container and transfer line/ion source are resistively heated to obtain the desired temperature, with currents up to 370 A for the transfer line/ion source and 1200 A for the target container. The target container and ion source are covered in Ta, W, and Mo heat screens, to have more uniform heat distribution during operations. The target container housing for the target material or external samples is made out of Ta material, due to its durability and refractory nature. Possible alternatives would be W or Re; however, due to the complex structures needed for target-unit assembly these materials are most difficult to manufacture accordingly. In cases where exposure to Ta should be limited, thin metal foil linings or covers such as 10–30 μm V and W foils are inserted in the target container. The target container’s inner diameter is 20 mm, and it is 200 mm long. The transfer line to the ion source is located in the middle of the target container perpendicular to the container.

The key accessory for molecular-beam formation is a gas injection line within the target unit, which is a two-piece Ta tube that connects the target-unit base with the target container. The gas injection line faces the transfer line on the target container and is connected via a hollow boron nitride (BN) cylinder to ensure electrical insulation. To control gas flow to the target container, 316 L grade sintered stainless-steel powder calibrated gas leaks (High Technology Products Ltd., East Sussex, UK) are installed on the base plate at the beginning of the gas line. Typical leak rates used are within the range of 4 $\times \;10^{-6}$ to 2 $\times \;10^{-4}$ mbar.L/s (for air). By controlling the gas pressure applied to the gas leak from the reservoir side, the gas flow into the target container can be varied.

To ionize the created gaseous atoms and molecules within the target container, various ion source and transfer-line modifications have been developed, tested, and used [21,56,57]. The most simple one of them is the surface ion source, which is a hot hollow metallic tube with a high work function ϕ, such as Ta (ϕ = 4.0–4.8 eV), W (ϕ = 4.3–5.2 eV), or Re (ϕ = 4.7–5.1 eV). They are typically operated at 2000–2400 °C and capable of ionizing low-Ionization Potential (IP) species with IP < 6 eV [21,58,59,60,61]. Laser ionization is also possible at MEDICIS, thanks to a dedicated laser laboratory, MELISSA (MEDICIS Laser Ion Source for Separator Assembly) [62,63]. The ionization of mono-atomic Sc was tested with different target-unit configurations (see Table 5), using a two-step laser scheme with auto-ionization resonance.

Because of the high mono-atomic Sc and molecule ionization or dissociation potential, the Versatile-Arc-Discharge Ion Source (VADIS) was mainly used in this study. VADIS is a Forced-Electron-Beam-Induced Arc Discharge (FEBIAD) or the so-called plasma ion source. It is an optimized version with an increased active volume and a reduced amount of graphite compared to its predecessor MK series [64]. In this study, the VD-5 target-unit type was most commonly used. It has a hot Ta transfer line from the target container to the VADIS ion source. The VADIS Ta cathode is directly connected to the transfer line and, hence, they are heated together to the same temperatures. Electron emission and acceleration through a grid into the anode body create plasma conditions upon electron impact with gaseous species at 1900–2100 °C. The electrons are deflected by an axial magnet on helical orbits. Because of the electron acceleration at 50–200 V (0–300 V total range) on the anode, the ion source is capable of ionizing/dissociating all low and high IP species reaching the ion source. Open Ta surfaces in one of the prototype target units were decreased by lining the transfer line and the cathode with tungsten. An open VADIS VD-5 target unit schematic overview is shown in Figure 4.

### 4.2. Molecular Ion Beams

Refractory and rare earth elements such as Sc are difficult to extract from thick ISOL target units in the form of mono-atomic gas [27]. Sc is known to form halides that are more volatile than the atomic counterpart and could, therefore, be used for extraction from target units in a molecular form. To create scandium-halide molecules, reactive gases such as carbon tetrafluoride (CF${\;\;}_{4}$, 99.995% pure), nitrogen trifluoride (NF${\;\;}_{3}$, 99.99% pure), and chlorine (Cl${\;\;}_{2}$, 99.8% pure) were applied to the target container via the calibrated gas leak.

Molecular and mono-atomic Sc ion beam formation is mainly obtained through stable molecule dissociation rather than direct ionization, because the dissociation potential of ScF${\;\;}_{x}\left(x=1-3\right)$ species is lower than their direct ionization [65,66]. Both the dissociation and ionization potential of atomic Sc and ScF${\;\;}_{x}$ molecules is >6 eV, which exceeds the work function of Ta, W, and Re, suggesting that the surface ion source is not suitable for Sc mass separation. Nevertheless, Sc${\;\;}^{+}$ and ScF${\;\;}^{+}$ beams have been reported at ISOLDE with a W surface source [36], due to certain released molecule-fraction dissociative ionization and drift electron acceleration in the extraction field. Hence, Sc mono-atomic ionization with a laser or halide-molecule ionization with a FEBIAD-type ion source should be preferred [65]. Since laser ionization is not efficient with a VADIS ion source, due to distance and colder ion source structures in between the transfer line and the extraction electrode, W and Re surface ion sources were used for laser resonant ionization tests. For VADIS ion source ionization efficiency tests and comparisons a mix of noble gases (He, Ne, Ar, Kr, Xe of 20% each, 99.999% pure) was also used.

### 4.3. Laser Resonant Ionization Results

Laser ionization tests were performed, using a stable ${\;\;}^{45}$Sc${\;\;}_{2}$O${\;\;}_{3}$ sample, which was dried on Ta foil and loaded into an empty target container. Resonance laser ionization of Sc isotopes is only possible for mono-atomic scandium vapor. The obtained response to the laser ionization showed that a chemical reduction and dissociation of the oxide molecule in the Ta target container had happened.

**Figure 5:**
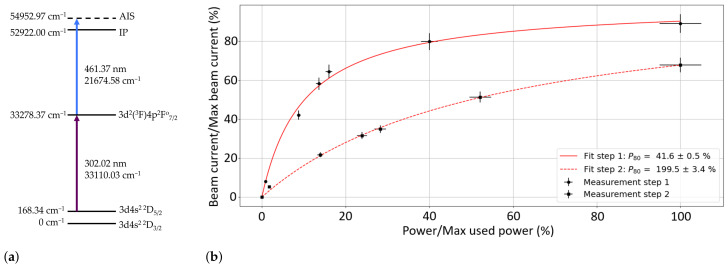
Mono-atomic Sc laser ionization scheme and power-saturation curve: (**a**) Two-step Sc laser ionization scheme used at MELISSA. (**b**) Laser power saturation for Sc ionization. Data were fit with $\frac{I}{I_{0}}=\frac{P/P_{sat}}{1+P/P_{sat}}$. X-axis: Power as a fraction of the nominal operating power during the efficiency tests.

Laser ionization was performed, using a two-step resonant ionization scheme, shown in Figure 5a, which was developed at AG LARISSA, Institute of Physics, Johannes Gutenberg University Mainz, Germany. The first-step laser excited the Sc from the 168.34 cm^−1^ state, for which the relative population was calculated to be 57.4% at 2000 °C, to the 33278.37 cm^−1^ energy level determined from [67]. The laser light for this step (302.02 nm) was generated using a titanium/sapphire (Ti:Sa) laser with an external frequency-tripling unit [68]. The light for the second step (461.37 nm) was produced using an intra-cavity doubled Ti:Sa laser. A scan of the second ionization step in the range [21600.00–22400.00 cm${\;\;}^{-1}$] was first performed to determine the Auto-Ionization State (AIS) that gave the highest ion current. Several AISs were measured and compared, with the strongest observed at an energy of 54952.97 cm^−1^. The second-step laser was operated at this frequency for the efficiency test. The power of the lasers during the efficiency test was 25 mW and 225 mW for the 302.02 nm and 461.37 nm light, respectively. Figure 5b shows the saturation curve of both excitation steps. The parameter $P_{80}$ is the laser power of the step that is required to observe 80% of the maximum-possible achievable laser ion current and is taken as the power at which the transition is considered saturated. In this case, step 1 was well-saturated, whereas step 2 had not reached saturation. With a higher power for step 2, the laser ion beam current could be improved by a factor of up to ×1.5.

During the tests, mono-atomic laser ionized Sc ions were observed when the target container temperature was increased over 1530 ± 10 °C for two different target units with a transfer-line temperature of 2000 °C. One of the target units was equipped with Re and the other with a W surface ion source. To obtain the relevant beam current for the Sc collection, the target-container temperature had to be increased over 2000 °C. This temperature well exceeds the metallic-foil target-material melting points that are considered suitable for radioactive Sc production, making it almost impossible to collect atomic Sc with high efficiency and within a reasonable collection time. In addition, from a target unit with a W transfer line/surface source, from a stable sample of 600 nAh (1 μg), 0.38 nAh were detected on the separated-beam Faraday cup over 2 days, corresponding to a collection efficiency of around 0.06%. This is the lower estimation of efficiency, and the actual value should be measured in future research.

The same ionization scheme was tested for the ionization of Sc with an irradiated ${\;\;}^{nat}$V-foil target unit. The target unit used for this experiment was equipped with the back of the transfer-line-heated container and the VADIS ion source. This is a target container design where the transfer-line heating connection is attached to the back of the line instead of the usual middle section. It creates a more homogeneous temperature profile along the whole length of the transfer line. Even though the VADIS ion source is not the best fit for laser ionization, atomic ${\;\;}^{45}$Sc${\;\;}^{+}$, ${\;\;}^{46}$Sc${\;\;}^{+}$, and ${\;\;}^{47}$Sc${\;\;}^{+}$ were observed with laser resonance when the target container temperature of 1570–1700 °C was achieved. After one day of operation, mono-atomic laser ionized species were no longer observed, even by increasing the target-container temperature, indicating full release of the Sc isotopes from the target material in an atomic form. Laser ionization tests of Sc were also performed with a target material fluorinated prior to irradiation; however, no laser resonance and mono-atomic Sc presence was observed with the same operating conditions.

### 4.4. Target and Ion Source Unit Development Results

In order to improve the conditions for the molecular beam formation and the temperature profile of the target container and to reduce Sc adsorption on target unit structures, multiple parts of the VADIS VD5 target units were modified and tested, as indicated in Figure 4 and summarized in Table 5.

**Figure 6:**
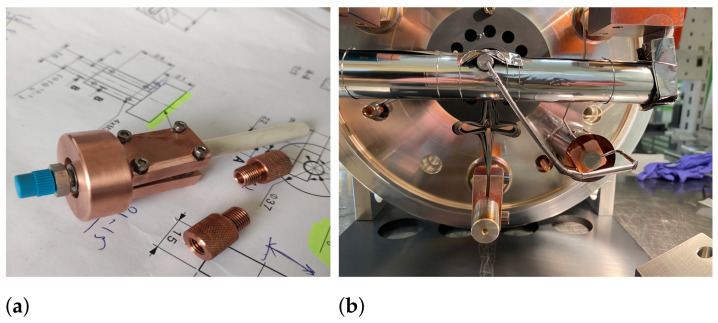
Gas-leak and BN-insulator Cu cooling structure for target unit: (**a**) Gas-leak cooling structure components. (**b**) Target unit with installed gas-leak cooling structure.

The calibrated gas leaks were identified as a weak point for target units when NF${\;\;}_{3}$ reactive gas was used for molecular-beam studies. The stainless steel melting point is in the range of 1375–1400 °C. However, the sintering of conventional powders is expected at about 50–80% of the material melting point ($T_{m}$) [23]. The radiation heat from the hot target container has been observed to reach over 340 °C during operations at the actively cooled base plate. Furthermore, the Ta gas tubing is directly connected to the target container and can conduct heat directly to the gas leak. This is sufficient to induce a chemical reaction with the NF${\;\;}_{3}$ gas and components of the stainless steel, such as iron, chromium, nickel, and molybdenum, leading to a reduced gas-leak rate and an increase in the total beam background. In addition, the gaseous particles from a hot target container can migrate towards the gas leak, where they condense on the surface of stainless steel or BN insulators. On the other hand, during the use of the Cl${\;\;}_{2}$ gas, degradation of the gas leak and an increase in the leak rate were observed. To reduce the possibility of reactions with the gas leak, a copper cooling structure was developed, which had strong direct contact with the gas leak, a BN insulator, and an actively cooled base plate (see Figure 6). Because of the perpendicular connection for Ta tubes to the BN insulator, it could also act as a cold trap to condense a fraction of outgassed particles and prevent them from depositing on the gas leak. During operation with the cooling structure and target container temperature at 1600 °C the insulator temperature still reached 110 °C.

Compared to previous target units, the additional cooling structure slightly reduced the TaF and TaO${\;\;}_{x}$F${\;\;}_{y}$ relative beam intensities in the total beam; however, it eventually did not prevent the gas leak from clogging when NF${\;\;}_{3}$ gas was applied to the target container at temperatures over 1400 °C. The leak rate reduced to a point where almost no fluorination of the target material was observed. When NF${\;\;}_{3}$ was used for stable ${\;\;}^{nat}$Ti metallic foil tests, the formation of titanium nitride was observed, suggesting relatively stable nitride species formation in the target container. Due to these observations, CF${\;\;}_{4}$ was used as a fluorinating agent.

**Figure 7:**
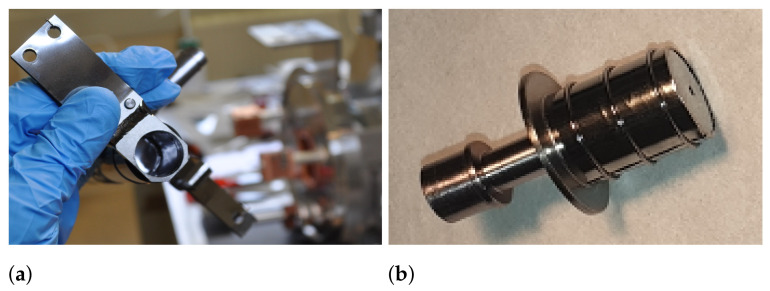
Target container and ion source cathode covered with metallic foils: (**a**) V lining in the target container. (**b**) VADIS cathode covered with W.

Sc has high adsorption enthalpy on a Ta surface (−521 kJ/mol [69]), of which the VADIS VD-5 target container, transfer line, and ion source are made. The effusion time from the target container can be approximated through delay-time estimation [70]:

(1) $$\tau_{\nu }=\frac{1}{\nu }=n_{wc}\left(\tau_{a}+\tau_{f}\right),$$

where $\tau_{\nu }$ [s${\;\;}^{-1}$] is the effusion mean delay time [s], ν [s${\;\;}^{-1}$] is the effusion time constant, n${\;\;}_{wc}$ [dim] the number of wall collisions, $\tau_{f}$ [s] is the mean flight time between two wall collisions, and $\tau_{a}$ [s] is the mean sticking time for a wall collision, which depends on the Frenkel equation [71]:

(2) $$\tau_{a}=\tau_{0}e^{\frac{-\Delta H_{a}ds}{RT}},$$

where $\tau_{0}$ [s] is the period of oscillation perpendicular to the surface, and where $\Delta H_{a}$ [Jmol${\;\;}^{-1}$] is the adsorption enthalpy, which depends on the element–surface combination number of collisions. By considering 1 $\times \;10^{5}$ collisions of mono-atomic Sc with Ta surfaces in the target container and transfer line, the time for Sc release from the target unit is then calculated to be ∼24 h with the Ta surface at 1550 °C. Here, re-adsorption on Ti and V has not been taken into account and, therefore, the real delay time could be even higher. In the case of W as the main surface material ($\Delta H_{a}$ = −507.3 kJ/mol [69]), the mean effusion time would drop to ∼11 h with the same conditions. To reduce Sc interactions with Ta surfaces and delayed release, the lining of the target-unit transfer line and coverage of the ion source cathode was performed with 25 μm-thick W foil and the target container with 25 μm-thick V foil (the same as the target material used for that particular target unit), as shown in Figure 7. Tungsten was used for the cathode and transfer line because the VADIS VD-5 ion source optimal operation temperature is ∼2000 °C. The reduced exposure to Ta structures in comparison to the regular Ta target container and transfer line, however, did not noticeably influence the release of the elemental Sc, the extraction onset temperature or the intensity of the ${\;\;}^{47}$ScF${\;\;}_{2}^{+}$ molecular beam, suggesting that mono-atomic Sc migration within the target unit is not the limiting release cause and could be attributed to interdiffusion or chemical diffusion.

**Figure 8:**
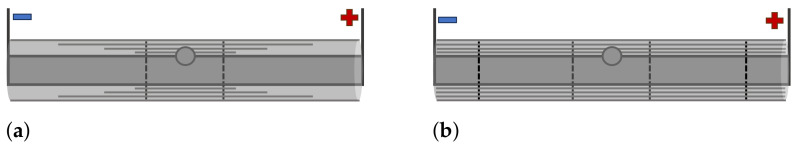
Heat screen placement on an ISOLDE/MEDICIS target container: (**a**) Prototype heat screen assembly. (**b**) Standard heat screen assembly.

Due to operation at high temperatures, the target container as well as the transfer line are shielded with multiple layers of heat screens. These help to maintain the heat inside the target container, achieve higher temperatures, and improve the homogeneity. Many studies have been done to homogenize the temperature profile in target containers for ISOL experiments [25]. Typically, the extremities of the target container would be 100–150 °C colder than the center part. This can be the limiting factor for radionuclide release because of their vapor condensation at the colder parts of the container. In fact, the transfer line has a temperature gradient and is not homogeneous throughout its full length. If radionuclide release from the target unit is close to the melting point of the target material, further heating and extraction of such radionuclides from the colder target-container extremities is not possible. If decreasing temperature profiles are applied from extremities towards the middle section of the container, migrating species might be forced to travel across the decreasing temperature profile towards the ion source. For these reasons, a new target-container type with transfer-line heating from the backside of the target container [25] was tested in combination with a prototype heat screen assembly (see Figure 8a). The prototype was expected to reduce the temperature difference between the extremities and the middle part of the target container as well as to improve the transfer-line temperature homogeneity. Typically, heat screens of Ta, Mo, and W are assembled along the full length of the target container and separated/secured with Ta wire with ⌀=1 mm (see Figure 8b). To heat the container, current flows from one side of the container to the opposite. It is postulated that in the prototype design the electric contact with the resistively heated container and heat screens is reduced, therefore increasing the temperature at the extremities of the container body.

The prototype heat screen assembly together with the back-of-line-heating target container did not meet the expected temperature gradient. Due to the necessity to heat the transfer line/cathode to 1900–2000 °C, the middle section of the target container remained higher than the extremities. Therefore, the heat screens from the transfer-line attachment to the target container had to be locally removed. This resulted in the quarterly section remaining the hottest and closest to the target-material melting point during nominal Sc-release temperature conditions. Deep plugs could be inserted in the target container from both opening sides up to the target material, by mitigating the gaseous-particle access to colder parts. In such a case, particle migration to the coldest target-container sections would be excluded, but the available volume for target material would be reduced.

Multiple modes of failure were observed for the target units and their modifications, mainly the VADIS ion source anode-voltage short circuit, due to the coating of the insulators. In the majority of cases, the target units were repaired or manipulated in conditions to reduce the coating until satisfactory drain-current and voltage-stability conditions were reached, to continue with the required tests or collections.

## 5. Target Material Developments and Sc Mass Separation at MEDICIS

Target units with empty containers were produced and first used in stable-beam tests. The target units were then loaded with prepared target materials and used for radioactive Sc production, release, and mass separation in multiple batches. In this study, the focus was set on medium-energy cyclotron-compatible and mass separator-compatible target materials, to investigate conditions and their potential for mass separation. The summary list of investigated target materials and produced charges is presented in Table 6.

### 5.1. Target Materials for Sc Ion Beams at MEDICIS

**Figure 9:**
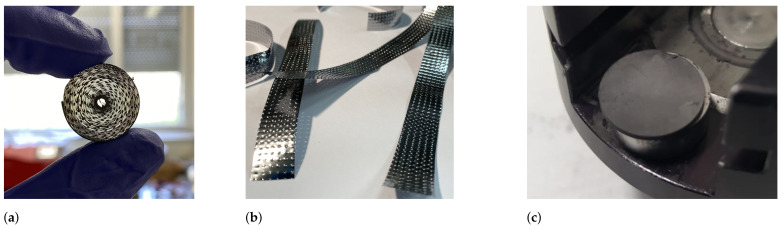
Embossed ${\;\;}^{nat}$Ti and ${\;\;}^{nat}$V metallic foils and pressed ${\;\;}^{nat}$TiC pills used for target charges: (**a**) ${\;\;}^{nat}$Ti double-foil roll. (**b**) Embossed metallic ${\;\;}^{nat}$V foil. (**c**) Pressed ${\;\;}^{nat}$TiC pill.

The metallic ${\;\;}^{nat}$Ti and ${\;\;}^{nat}$V target charges were made out of thin foils that were cut into 10–15 mm-wide strips, rolled in a cylindrical shape (⌀<20 mm) and held together with a Ta wire (see Figure 9a). To reduce sintering, the foil strips were embossed, creating a hill–valley pattern and a larger space in between the rolled layers (see Figure 9b). To increase the production yield from irradiation, ${\;\;}^{nat}$Ti double-layer foil rolls were prepared, with one strip layer embossed and the second kept flat. The 25 μm-thick metallic ${\;\;}^{nat}$Ti and ${\;\;}^{nat}$V foils were purchased from Goodfellow Cambridge Ltd. (Huntingdon, UK). The rolls were washed in 70 % ethanol and an ultrasonic bath for 10 min and dried in air.

**Figure 10:**
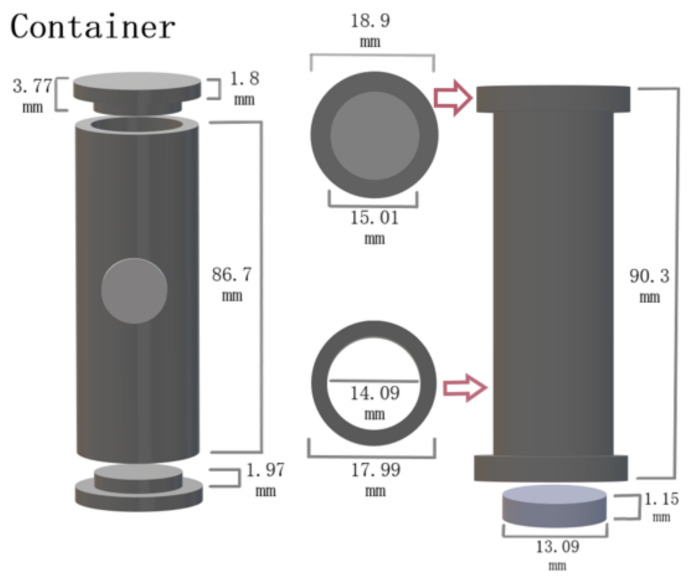
Carbon sleeve and ${\;\;}^{nat}$TiC pellet dimensions used for target charge production.

Titanium carbide ${\;\;}^{nat}$TiC powder with a particle size of 1–2 μm (bought from Alfa Aesar, US) was pressed in 13 ± 1 mm diameter and 1.2 ± 0.2 mm-thick-sized compact cylindric pellets, weighing approximately 0.42 g each (see Figure 9c). The measured specific surface area was 2.48 m${\;\;}^{2}$g${\;\;}^{-1}$. A semi-automated 15-ton hydraulic press (Specac Ltd., Orpington, UK) was used to press the ${\;\;}^{nat}$TiC powder in pellets with 295–330 MPa pressure. The prepared pellets were loaded in a carbon sleeve (see Figure 10) to maintain their shape and integrity during transfer, outgassing, irradiation, mass separation, and to ease the loading of the charge inside the target container. The carbon sleeve had a circular opening in the middle to allow effusing particles to escape. After the pellets were loaded into the carbon sleeve, they were placed in a separate target unit that consisted of only the base plate, target container, and heat screens. The ${\;\;}^{nat}$TiC pellets with the carbon sleeve were outgassed overnight at 1800 °C temperature, with the pressure of the target unit being kept below 1.5 $\times \;10^{-5}$ mbar. After outgassing, a few pellets were removed for characterization and weight-loss measurements. The target charge was then loaded inside the target container exactly at the middle with the sleeve opening facing the transfer line to the ion source.

### 5.2. Molecular-Beam Development Results

To investigate the stability and release of Sc radionuclides from irradiated MEDICIS target units in the form of mono-atomic and molecular ions, a series of experiments were done with the Offline-1 mass separator. Halide, oxide, and oxyhalide molecular ion formation and release were investigated with the VADIS ion source because of the molecule’s high ionization or dissociation potential. To assess the target-material influence on total and separated beams as well as on molecule formation, ${\;\;}^{45}$Sc molecular-beam experiments were performed with and without the natural target materials of ${\;\;}^{nat}$Ti (${\;\;}^{46}$Ti-8.25%, ${\;\;}^{47}$Ti-7.44%, ${\;\;}^{48}$Ti-73.72%, ${\;\;}^{49}$Ti-5.41%, ${\;\;}^{50}$Ti-5.18%) and ${\;\;}^{nat}$V (${\;\;}^{50}$V-0.25%, ${\;\;}^{51}$V-99.75%) being present in the target container.

Scandium halides and especially fluorides were chosen as the molecules of highest interest, due to halide-molecule volatility, the mono-isotopic nature of fluorine, the absence of scandium oxyhalides, stability at relatively high temperatures, and practical aspects of handling reactive chemicals and gases. Various chemical forms of stable ${\;\;}^{45}$Sc isotope compounds with known concentrations and reactive/buffer gases were used in this study. Anhydrous, crystalline ${\;\;}^{45}$ScF${\;\;}_{3}$ (>99.9% purity) was purchased from Appollo Scientific (Manchester, UK), anhydrous, crystalline ${\;\;}^{45}$ScCl${\;\;}_{3}$ (>99.9% purity) from Fisher Scientific AG (Schwerte, Germany), and ${\;\;}^{45}$Sc${\;\;}_{2}$O${\;\;}_{3}$ in 5 % HNO${\;\;}_{3}$ (>99.995% purity, 1000 μg/mL) was purchased from Alfa Aesar (Ward Hill, MA, USA). All the liquid samples were dried on metallic Ta, ${\;\;}^{nat}$Ti, or ${\;\;}^{nat}$V substrates in the air at 70–80 °C, before being loaded inside the target container. To determine the molecular-beam feasibility for Sc mass separation, an extraction-efficiency measurement was done, resulting in ∼5 % efficiency for an ${\;\;}^{45}$Sc${\;\;}_{2}^{+}$ molecular beam from stable ${\;\;}^{45}$Sc${\;\;}_{2}$O${\;\;}_{3}$ and NF${\;\;}_{3}$ as the fluorine source in an empty target container.

Because of the reactive nature of fluorine also towards the target unit structures, the total beam extracted from target units holds a vast amount of high-intensity (few nA to μA) molecular and mono-atomic beams. In this study, attention was drawn only toward the beams directly produced from the target material and the ones overlapping the intended radioactive Sc molecular-beam atomic-mass regions. The observed atomic and molecular ions of interest from each stable sample are indicated in Table 7.

Doubly charged ions of both mono-atomic and molecular beams were observed, due to dissociation and electron-impact energy (50–200 eV). Oxygen-containing molecules are formed from native oxide layers and impurities of ${\;\;}^{nat}$Ti, ${\;\;}^{nat}$V, and ${\;\;}^{nat}$Ta target-unit structures. Due to the mono-isotopic nature of stable ${\;\;}^{45}$Sc, the distinction in mass spectra of a single, weak intensity line is often not conclusive and certain species were not confirmed. The intensities of such beams were also often too low to allow collection and post-analysis with Inductively Coupled Plasma Mass Spectrometry (ICP-MS). Radioactive isotope collections, however, did confirm or deny certain hypotheses. Sc was found to form scandium fluorides mainly, and no clear release was observed as oxyfluorides, chlorides, oxychlorides, and oxides.

High-intensity contaminant beams should be avoided, due to the possibility of coating insulating parts of the mass separator or VADIS ion source. Due to fluorination also, intense beams of CO${\;\;}^{+}$, BeF${\;\;}^{+}$, BeF${\;\;}_{2}^{+}$, and COF${\;\;}^{+}$ are formed in VADIS VD-5 type target units, contributing to the total beam. BeF${\;\;}_{2}^{+}$ and COF${\;\;}^{+}$ are creating isobaric contamination at mass 47 with relatively high beam intensities. Because of high-beam-current-induced sputtering [41], the collection of mono-atomic ${\;\;}^{47}$Sc${\;\;}^{+}$ or ${\;\;}^{47}$Ca${\;\;}^{+}$ is not favored using a VADIS ion source if target material is being fluorinated. Chlorine injections in the target units were only available at MEDICIS due to the absence of a specified gas injection and neutralization system at Offline-1. Cl${\;\;}_{2}$ tests were carried out on already-fluorinated and pre-irradiated target materials because, at the time of this study, only MEDICIS was licensed and permitted to handle Cl${\;\;}_{2}$ gas.

### 5.3. Sc Radionuclide Release and Mass Separation Results

The target materials proposed for Sc radionuclide production as well as the target units and their modifications were tested on Offline-1 and MEDICIS mass separators, for target-unit functionality, stable isotope beams, irradiated-material Sc radionuclide release, and mass separation. Before the start-up of the MEDICIS facility, the collection efficiency for Sc radionuclides from the available ISOLDE-yield database was estimated at 5% [54]; however, no Sc radionuclides have been collected since its first radioactive ion beams in 2017 [24]. The developments in this study resulted in the first mass separation and collection of isotopically pure ${\;\;}^{44g/m}$Sc, ${\;\;}^{46}$Sc, and ${\;\;}^{47}$Sc radionuclides, with radioactivity ranging from a few kBq up to a few MBq. For the first time at MEDICIS, Sc molecular-beam extraction and collection efficiencies exceeded 1% under controlled operation conditions from a micrometric ${\;\;}^{nat}$TiC target material.

In contrast to stable beam tests, radioactive ion beams (as with laser ionized Sc${\;\;}^{+}$) were only observed above temperatures of 1500 °C. This exposed the first main limitation of ${\;\;}^{nat}$Ti-foil use as ISOL target material—a relatively low melting/sublimation point (1668 °C). Diffusion of radionuclides out of the target material and their effusion as well as adsorption/desorption on target-unit structures is a thermally activated process. Therefore, ${\;\;}^{nat}$V metal or carbon stabilized ${\;\;}^{nat}$TiC with higher melting points were more suitable for mass separation. Another advantage of ${\;\;}^{nat}$TiC powder was its high specific surface area compared to metallic foils; ${\;\;}^{nat}$Ti or ${\;\;}^{nat}$V powders could also be used; however, sintering effects should be addressed to re-use the target units for multiple batches and to sustain the release over long-term operations.

Another observed limitation of all the tested target materials for mass separation is the target-material reactivity towards the halogenating agent and the release of impurities at elevated temperatures, especially for ${\;\;}^{nat}$V and ${\;\;}^{nat}$TiC. A MEDICIS mass separator together with a VADIS ion source is preferably operated up to a 10 μA beam range with a capacity of around 50 μA; therefore, even mg-amount impurities in the target material can cause delays in isotope collection for days, due to unsatisfactory ion-beam purity. The main impurity in V foils was Al, which reacted with fluorine and formed volatile μA-range intensity AlF${\;\;}_{x}^{+}$ (X = 1–3) beams, suppressing the Sc fluoride molecule formation. After several days of operations, the reduction of Al release was not definitively observed. TiC, on the other hand, was releasing a few-hundred nA-range Cr${\;\;}^{+}$ beam at temperatures above 1800 °C, which was delaying further heating and increase of the Sc collection rate. In contrast to Al in V, the Cr-beam intensity reduced over time.

**Figure 11:**
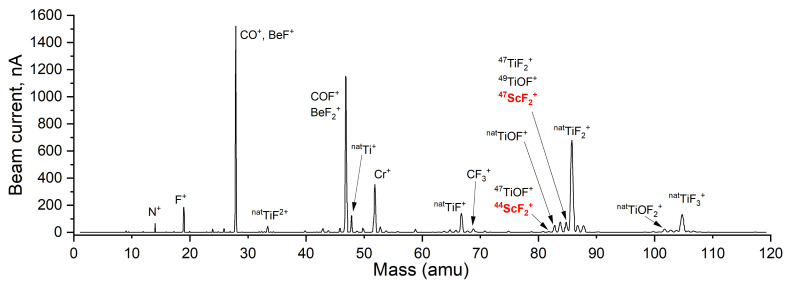
Mass scan of irradiated and fluorinated ${\;\;}^{nat}$TiC target material with ion source at 2000 °C and target container at 2000 °C.

**Figure 12:**
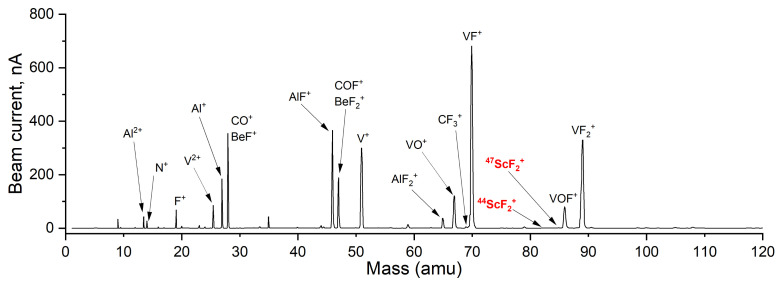
Mass scan of irradiated and fluorinated ${\;\;}^{nat}$V target material with ion source at 2000 °C and target container at 1725 °C.

Mass scans of fluorinated ${\;\;}^{nat}$TiC and ${\;\;}^{nat}$V foils at various operating temperatures with indicated main chemical species in total beam up to amu 120 are shown in Figure 11 and Figure 12. Occasionally, mass scans up to 260 amu (at Offline-1) and 230 amu (at MEDICIS) were also taken, to monitor tantalum, tantalum oxide, tantalum fluoride, and tantalum oxyfluoride beams, which also act as a probe for fluorination and fluorine saturation within the target unit. The beam intensities between the two mass scans at Figure 11 and Figure 12 have a noticable difference and are not comparable, due to each being taken with different target units, different target-container temperatures, ion source parameters, ionization efficiencies, and amounts of applied CF${\;\;}_{4}$ gas. Nevertheless, they provide an understanding of the main chemical species present in the total beam. The total beam composition for ${\;\;}^{nat}$Ti is similar to ${\;\;}^{nat}$TiC, due to Ti, TiO${\;\;}_{2}$, and stable TiC reactivity towards the halogenating gases at elevated temperatures. Also, the intense Cr beam is absent with ${\;\;}^{nat}$Ti-foil target materials and more intense titanium oxyfluoride beams are present, due to the native oxide layer on metallic Ti foil. The Cr absence can be attributed to the lower operated temperatures of the ${\;\;}^{nat}$Ti-foil target materials. For ${\;\;}^{nat}$V target material, the background of the Sc molecular-beam mass region is significantly lower than ${\;\;}^{nat}$Ti or ${\;\;}^{nat}$TiC as no vanadium fluoride or oxyfluoride side bands are overlapping the ScF${\;\;}_{2}^{+}$. This suggests ${\;\;}^{nat}$V as more suitable for reduced isobaric background Sc molecular-beam collection and also presents the potential for high-melting-point-VC or VB investigation as target materials. Ti or V isotopes from the collection final product can be removed with chemical separation methods, such as ion-exchange column chromatography.

## 6. Discussion

The production cross sections of Sc radionuclides from ${\;\;}^{nat}$Ti and ${\;\;}^{nat}$V target material irradiated with high-energy protons (>1 GeV) are in the same 10–25 mb range as the ones achievable with medium-energy cyclotrons. Although the beam intensity used for medical-isotope production of the CERN PSB is lower than a typical cyclotron, the production yield at MEDICIS is compensated with the target charge thickness, due to the available high-proton energy. Because of proton-beam straggling that travels through the ISOLDE UC${\;\;}_{x}$ target charge, the radionuclide production yield could benefit from larger diameter and volume target containers in the case of indirect irradiation. A larger-diameter target-container prototype was proposed and is being developed at MEDICIS [54]. With the combination of efficient release and sufficient irradiation times, MEDICIS can achieve clinical doses of Sc radionuclides comparable to the ones that can be achieved by medium-energy cyclotrons in medical or research centers (see Table 4). Furthermore, MEDICIS has the potential to produce and deliver high molar activity and isotopic-purity Sc radionuclides, thanks to the mass separation technique and parallel-collection possibility for simultaneous ${\;\;}^{44g/m}$Sc/${\;\;}^{47}$Sc collection from a single batch. An increase in cyclotron target charge thickness and irradiation times can increase the yield; however, Sc isotope contaminant buildup is inevitable. The dissolution of metallic Ti foils, TiO${\;\;}_{2}$, or TiC after irradiation is extremely difficult, whereas mass separation would omit this step as well. Therefore, Sc production from economically feasible target materials should include a mass separation step to reach a purity level high enough for medical applications. In contrast to higher isotopic enrichment for cyclotron targets, mass separation requires higher-chemical-purity materials for better release, mass separation conditions, and mass separator longevity.

For Sc production with cyclotrons and mass separation, irradiated high-activity samples can be transferred into the target units in a hot cell or by designing upcoming mass separator facilities with a dedicated beamline for an online-isotope-production approach. In the latter case, the target unit and charge could be conditioned in a vacuum and outgassed just before irradiation and mass separation, irradiated in a vacuum, and a higher amount of short-lived Sc radionuclides mass separated. The MEDICIS mode of operation at present has certain limitations for experiment preparation as well as for the isotope collection, by requiring a few hours to reach operational conditions (temperature, pressure, extraction voltage) and allowing for other volatile species to outgas from the irradiated target material. The batch-mode production also allows for only a few parameter investigations during a single experiment.

Laser ionization is the best way to create very pure Sc ion beams and presents huge potential, as it is element-selective and in combination with mass separation could save a lot of time that is required for chemical post-purification. However, the obtained collection efficiency is too low for significant activity collections. The low efficiency could be explained by Sc molecule formation with target-unit structures, target-material impurities, and molecule stability in high temperatures. Without further understanding of Sc release from target materials and ISOL target units, lasers are not the preferred ion source. To achieve a higher collection efficiency, the target container has to be heated above 2000 °C, but at such temperatures only B- or C-stabilized materials such as TiC or TiB could be used. However, without the target-material fluorination, Sc would be instead stabilized as, e.g., ScC and very little or no mono-atomic Sc eventually released [20,28].

The observed mono-atomic Sc${\;\;}^{+}$ ions from stable-beam experiments show a chemical reduction and dissociation of the oxide molecule in the Ta environment, even though the ${\;\;}^{45}$Sc${\;\;}_{2}$O${\;\;}_{3}$ with low vapor pressure and a high melting point is a stable molecule even at high temperatures and reacts with Ta only at 2250 °C. Sc${\;\;}_{2}$O${\;\;}_{3}$ can undergo a reduction in the presence of C or CO, which are found in the target units either from impurities in Ta structures (for surface source targets) or VADIS ion source support and assembly, as shown in Figure 6b. ScO is, in fact, the most stable diatomic gaseous scandium molecule known, with a dissociation energy of 674 kJ/mol (6.99 eV) [72]; therefore, dissociation by surface interaction is not expected.

Even though laser ionization has a huge potential, thus far the best method of Sc extraction from target units is through volatile molecule formation. For volatile scandium-molecule formation, fluorine is the preferred choice to maximize the collection yield, because of its stable fluoride mono-isotopic nature. Chlorine, on the other hand, has two stable naturally occurring isotopes of amu 35 and 37. The double-collection possibility at MEDICIS offers to solve this issue, since the mass difference is only 2 amu. However, Cl${\;\;}_{2}$ is not suitable in order to maximize on ${\;\;}^{44g/m}$Sc collection as, therefore, isobaric contaminants of ${\;\;}^{46}$Sc${\;\;}^{35}$Cl${\;\;}^{+}$ interfere with ${\;\;}^{44g/m}$Sc${\;\;}^{37}$Cl${\;\;}^{+}$ collection at 81 amu sideband. The ionization efficiency of molecules is another limiting factor, especially in high-total-beam-current conditions. For this reason, too-rapid release from the target material is not desired and, rather, extended collection would be preferred. To explain Sc release from target units, further investigations in molecular-beam formation mechanisms and dynamics are required. Since target materials as well as target-unit structures are always accompanied by native oxides, Sc release in an oxidative environment must be better understood. Also, more control over temperature in the transfer line for VADIS VD-5 target units could be beneficial, by having the ability to condense unwanted species before they reach the ion source. Although the mass separator is capable of delivering isotopically pure radionuclides, certain isobars from the target material and target units are also collected. To obtain high-molar-activity Sc radionuclides a radiochemical separation step is still performed afterwards [73,74]; however, this is a subject for another study.

The preferred target material was ${\;\;}^{nat}$TiC, in terms of operation at high temperatures and providing the highest yield of mass separated Sc radionuclides in this study. Even though the TTY for ${\;\;}^{nat}$TiC target charge was lower, the better results could be explained by the carbon stabilization, restricting too-rapid release and, at the same time, CF${\;\;}_{4}$ etching of the material, as well as the higher specific surface in comparison to metallic foils. Nanoscale ${\;\;}^{nat}$TiC can reach a few-orders-of-magnitude-higher specific surface area [23]. Counterintuitive to ScC stabilisation, nanoscale ${\;\;}^{nat}$TiC and carbon- or boron-stabilized vanadium target materials with a molecular-beam approach for mass separation should be investigated in future studies.

## 7. Conclusions

Cyclotron-suitable target materials for medical Sc radionuclide production—such as ${\;\;}^{nat}$Ti, ${\;\;}^{nat}$V metallic foils, and ${\;\;}^{nat}$TiC target materials—were used at CERN-MEDICIS for the first time. An Sc-radionuclide-production thick-target yield with 1.4 GeV protons at MEDICIS is equivalent to a medium-energy-cyclotron production, high enough to achieve clinical doses and benefit from the mass separation of ${\;\;}^{46}$Sc and${\;\;}^{46}$Sc.

Mono-atomic Sc isotopes were obtained at MEDICIS for the first time with a new two-step laser resonance ionization scheme; however, the estimated collection efficiency (0.06%) is lower than with the molecular beam approach. The target container and materials must be heated to temperatures above the metallic ${\;\;}^{nat}$Ti- and ${\;\;}^{nat}$V-foil melting points to obtain feasible collection efficiencies. Laser resonance was not observed with a pre-fluorinated target material, suggesting that fluorine presence prevents Sc release from the target unit in a mono-atomic form. Nevertheless, laser ionization could serve as a control tool to verify and study Sc radionuclide release from metallic-foil target materials before molecular-beam formation.

Sc release and mass separation at MEDICIS is most suitable with target materials that could be operated above 1530 °C. In terms of radionuclide production, high operating temperatures, and stability, ${\;\;}^{nat}$TiC is the most suitable target material for molecular-Sc-beam mass separation. The sustained and controlled release of Sc radionuclides from fluorinated high-specific-surface-area ${\;\;}^{nat}$TiC target material shows potential in nanoscale-target-material investigations. More selective and temperature-controlled target and ion source unit designs are necessary to reduce isobaric impurities, promote the desired molecule formation, and transfer to the ion source during mass separation.

High molar activity and radiochemical purity ${\;\;}^{44g/m}$Sc, ${\;\;}^{46}$Sc, and ${\;\;}^{47}$Sc radionuclides were successfully extracted and mass separated at CERN-MEDICIS as molecular difluoride ion beams with collection efficiencies of more than 1% from micrometric ${\;\;}^{nat}$TiC. Stable and sustainable operation conditions to produce medically important Sc by mass separation were obtained. Mono-atomic Sc adsorption on the Ta target-unit structures was found not to be the limiting release cause, suggesting possible interdiffusion (chemical diffusion) effects that will be investigated in further studies. The conditions of Sc radionuclide release from metallic ${\;\;}^{nat}$Ti foils and molecular-beam formation mechanisms also have to be further investigated. The achieved collection efficiency, yield, purity, and radiochemical separation will be the subject of another article.

## Figures and Tables

**Table 1:** Most common production reactions of medical Sc radionuclides.

Radionuclide	Reaction	Max Cross Section	Sc Isotope Impurities	Mass Separation	Note
${\;\;}^{43}$Sc	${\;\;}^{43}$Ca(p,n)${\;\;}^{43}$Sc	300 mb	${\;\;}^{44g}$Sc	Not needed	<0.14% ${\;\;}^{43}$Ca in ${\;\;}^{nat}$Ca, max enrichment 90% [8,11,12].
	${\;\;}^{44}$Ca(p,2n)${\;\;}^{43}$Sc	170 mb	${\;\;}^{44g}$Sc, ${\;\;}^{44m}$Sc	Needed	${\;\;}^{44m}$Sc cannot be avoided [8,13].
	${\;\;}^{46}$Ti(p,α)${\;\;}^{43}$Sc	45 mb	${\;\;}^{44g}$Sc, ${\;\;}^{44m}$Sc	Preferred	[13,14].
${\;\;}^{44g}$Sc	${\;\;}^{nat}$Ca(p,n)${\;\;}^{44g}$Sc	10 mb	${\;\;}^{43}$Sc, ${\;\;}^{44m}$Sc, ${\;\;}^{46}$Sc *, ${\;\;}^{47}$Sc, ${\;\;}^{48}$Sc *	Needed	[8,13].
	${\;\;}^{44}$Ca(p,n)${\;\;}^{44g}$Sc	700 mb	${\;\;}^{44m}$Sc	Not needed	${\;\;}^{44m}$Sc can be minimized with lower proton energy, but not avoided. Enrichment of ${\;\;}^{44}$Ca > 99%. Very expensive [5].
	${\;\;}^{47}$Ti(p,α)${\;\;}^{44g}$Sc	70 mb	${\;\;}^{43}$Sc, ${\;\;}^{44m}$Sc, ${\;\;}^{46}$Sc	Needed	[13,15].
	${\;\;}^{44}$Ca(d,2n)${\;\;}^{44g}$Sc	540 mb	${\;\;}^{43}$Sc, ${\;\;}^{44m}$Sc	Needed	Require deuterons up to 20 MeV.
	${\;\;}^{45}$Sc(p,2n)${\;\;}^{44}$Ti/${\;\;}^{44g}$Sc	45 mb	-	Not needed	${\;\;}^{44}$Ti (T${\;\;}_{1/2}$ = 60 y). Expensive production.
${\;\;}^{47}$Sc	${\;\;}^{47}$Ti(n,p)${\;\;}^{47}$Sc	250 mb	${\;\;}^{46}$Sc	Needed	Requires fast neutrons (>1 MeV).
	${\;\;}^{46}$Ca(n,γ)${\;\;}^{47}$Ca/${\;\;}^{47}$Sc	700 mb	-	Not needed	${\;\;}^{47}$Ca (T${\;\;}_{1/2}$ = 4.5 days) as ${\;\;}^{47}$Sc generator [6,7,16]. 0.004% ${\;\;}^{46}$Ca in ${\;\;}^{nat}$Ca, max enrichment 30% [17]. Very expensive.
	${\;\;}^{48}$Ti(p,2p)${\;\;}^{47}$Sc	35 mb	${\;\;}^{46}$Sc	Needed	Requires up to 30 MeV energy proton cyclotrons. Max enrichment is 96%. ${\;\;}^{46}$Sc cannot be avoided [18].
	${\;\;}^{48}$Ca(p,2n)${\;\;}^{47}$Sc	800 mb	${\;\;}^{48}$Sc	Needed	0.19% ${\;\;}^{48}$Ca in ${\;\;}^{nat}$Ca, max enrichment 97 % [17].

* ${\;\;}^{46}$Sc (T${\;\;}_{1/2}$ = 83.79 d, E${\;\;}_{\gamma }$= 889; keV 1120 keV) and ${\;\;}^{48}$Sc (T${\;\;}_{1/2}$ = 43.71 h, E${\;\;}_{\gamma }$= 983 keV; 1037 keV; 1312 keV).

**Table 2:** Sc radionuclide release from target materials used at ISOLDE.

Target Material	Operated Temperature, °C	Release Conditions
${\;\;}^{nat}$Ti metallic foils	>1600	W surface ion source. Fluorination with CF${\;\;}_{4}$. Only Sc${\;\;}^{+}$ and ScF${\;\;}^{+}$ observed and target molten after mass separation [36].
${\;\;}^{nat}$TiC (1–50 μm)	1900	Slow release that did not increase by fluorination with CF${\;\;}_{4}$ [28].
	2300	No Sc released [31].
${\;\;}^{nat}$TiC-CNT (nanometric)	1500	No Sc released. [20].
${\;\;}^{nat}$TiC-CB (nanometric)	1500–1740	Re surface source. No Sc was released. [20].
${\;\;}^{nat}$V powder	1800	No Sc released [31].
${\;\;}^{nat}$VC (1–50 μm)	1900	Slow release that did not increase by fluorination with CF${\;\;}_{4}$ [28].
	2300	No Sc released. Higher other radionuclide release rates than from ${\;\;}^{nat}$TiC [31].

**Table 3:** In-target Sc radionuclide production yields from FLUKA.

Radionuclide	Target Material *	Direct HRS, Nuclei/g/Primary	Indirect HRS Nuclei/g/Primary	Indirect GPS ISIS Nuclei/g/Primary
${\;\;}^{43}$Sc	${\;\;}^{nat}$Ti	2.97 ± 0.05 $\times \;10^{-5}$	2.45 ± 0.15 $\times \;10^{-6}$	3.6 ± 0.2 $\times \;10^{-6}$
	${\;\;}^{nat}$V	1.81 ± 0.03 $\times \;10^{-5}$	1.5 ± 0.2 $\times \;10^{-6}$	2.19 ± 0.15 $\times \;10^{-6}$
	TiC	2.37 ± 0.03 $\times \;10^{-5}$	1.9 ± 0.2 $\times \;10^{-6}$	2.9 ± 0.2 $\times \;10^{-6}$
${\;\;}^{44g}$Sc	${\;\;}^{nat}$Ti	5.91 ± 0.09 $\times \;10^{-5}$	5.2 ± 0.3 $\times \;10^{-6}$	7.8 ± 0.2 $\times \;10^{-6}$
	${\;\;}^{nat}$V	3.67 ± 0.02 $\times \;10^{-5}$	3.0 ± 0.2 $\times \;10^{-6}$	4.67 ± 0.19 $\times \;10^{-6}$
	TiC	4.52 ± 0.07 $\times \;10^{-5}$	3.51 ± 0.19 $\times \;10^{-6}$	5.70 ± 0.18 $\times \;10^{-6}$
${\;\;}^{44m}$Sc	${\;\;}^{nat}$Ti	5.91 ± 0.09 $\times \;10^{-5}$	5.2 ± 0.3 $\times \;10^{-6}$	7.8 ± 0.2 $\times \;10^{-6}$
	${\;\;}^{nat}$V	3.67 ± 0.02 $\times \;10^{-5}$	3.0 ± 0.2 $\times \;10^{-6}$	4.67 ± 0.19 $\times \;10^{-6}$
	TiC	4.52 ± 0.07 $\times \;10^{-5}$	3.51 ± 0.19 $\times \;10^{-6}$	5.70 ± 0.18 $\times \;10^{-6}$
${\;\;}^{46}$Sc	${\;\;}^{nat}$Ti	7.00 ± 0.11 $\times \;10^{-5}$	5.83 ± 0.12 $\times \;10^{-6}$	9.1 ± 0.2 $\times \;10^{-6}$
	${\;\;}^{nat}$V	4.22 ± 0.08 $\times \;10^{-5}$	3.5 ± 0.2 $\times \;10^{-6}$	5.8 ± 0.3 $\times \;10^{-6}$
	TiC	5.407 ± 0.016 $\times \;10^{-5}$	4.22 ± 0.14 $\times \;10^{-6}$	7.22 ± 0.15 $\times \;10^{-6}$
${\;\;}^{47}$Sc	${\;\;}^{nat}$Ti	1.72 ± 0.02 $\times \;10^{-4}$	1.37 ± 0.07 $\times \;10^{-5}$	2.10 ± 0.05 $\times \;10^{-5}$
	${\;\;}^{nat}$V	5.89 ± 0.14 $\times \;10^{-5}$	4.4 ± 0.2 $\times \;10^{-6}$	6.7 ± 0.6 $\times \;10^{-6}$
	TiC	1.296 ± 0.013 $\times \;10^{-4}$	1.03 ± 0.04 $\times \;10^{-5}$	1.69 ± 0.06 $\times \;10^{-5}$
${\;\;}^{44}$Ti	${\;\;}^{nat}$Ti	1.85 ± 0.06 $\times \;10^{-5}$	1.21 ± 0.08 $\times \;10^{-6}$	2.2 ± 0.3 $\times \;10^{-6}$
	${\;\;}^{nat}$V	9.1 ± 0.6 $\times \;10^{-6}$	8.9 ± 0.9 $\times \;10^{-7}$	1.41 ± 0.18 $\times \;10^{-6}$
	TiC	1.39 ± 0.06 $\times \;10^{-5}$	1.35 ± 0.18 $\times \;10^{-6}$	1.68 ± 0.14 $\times \;10^{-6}$
${\;\;}^{47}$Ca	${\;\;}^{nat}$Ti	1.4 ± 0.3 $\times \;10^{-6}$	1.1 ± 0.9 $\times \;10^{-7}$	2.0 ± 0.2 $\times \;10^{-7}$
	${\;\;}^{nat}$V	1.5 ± 0.2 $\times \;10^{-6}$	1.5 ± 0.6 $\times \;10^{-7}$	1.5 ± 0.4 $\times \;10^{-7}$
	TiC	8.9 ± 1.5 $\times \;10^{-7}$	6 ± 3 $\times \;10^{-8}$	7 ± 4 $\times \;10^{-8}$

* Target charge density of 1.5 g/cm${\;\;}^{3}$ for ${\;\;}^{nat}$Ti and ${\;\;}^{nat}$V, and of 2.7 g/cm${\;\;}^{3}$ for ${\;\;}^{nat}$TiC was considered.

**Table 4:** Thick-target yields for typical MEDICIS and cyclotron targets.

Radionuclide	Target Material	MEDICIS, Bq/μAh	30 MeV Cyclotron, Bq/μAh	70 MeV Cyclotron, Bq/μAh
${\;\;}^{43}$Sc	${\;\;}^{nat}$Ti	3.0 $\times \;10^{9}$	1.5 $\times \;10^{7}$	4.9 $\times \;10^{7}$
	${\;\;}^{nat}$V	1.9 $\times \;10^{9}$	-	2.3 $\times \;10^{6}$
	TiC	1.6 $\times \;10^{9}$	7.3 $\times \;10^{6}$	2.3 $\times \;10^{7}$
${\;\;}^{44g}$Sc	${\;\;}^{nat}$Ti	6.0 $\times \;10^{9}$	2.0 $\times \;10^{8}$	2.3 $\times \;10^{8}$
	${\;\;}^{nat}$V	3.7 $\times \;10^{9}$	-	5.4 $\times \;10^{7}$
	TiC	3.0 $\times \;10^{9}$	1.1 $\times \;10^{8}$	1.1 $\times \;10^{8}$
${\;\;}^{44m}$Sc	${\;\;}^{nat}$Ti	4.4 $\times \;10^{8}$	4.2 $\times \;10^{6}$	7.5 $\times \;10^{6}$
	${\;\;}^{nat}$V	2.7 $\times \;10^{8}$	-	3.8 $\times \;10^{6}$
	TiC	2.2 $\times \;10^{8}$	2.3 $\times \;10^{6}$	3.6 $\times \;10^{6}$
${\;\;}^{46}$Sc	${\;\;}^{nat}$Ti	3.0 $\times \;10^{7}$	1.1 $\times \;10^{5}$	5.7 $\times \;10^{5}$
	${\;\;}^{nat}$V	1.8 $\times \;10^{7}$	2.4 $\times \;10^{-2}$	2.6 $\times \;10^{5}$
	TiC	1.5 $\times \;10^{7}$	6.3 $\times \;10^{4}$	2.7 $\times \;10^{5}$
${\;\;}^{47}$Sc	${\;\;}^{nat}$Ti	9.2 $\times \;10^{8}$	5.6 $\times \;10^{6}$	6.9 $\times \;10^{6}$
	${\;\;}^{nat}$V	3.2 $\times \;10^{8}$	1.5 $\times \;10^{6}$	2.9 $\times \;10^{6}$
	TiC	4.5 $\times \;10^{8}$	3.0 $\times \;10^{6}$	3.3 $\times \;10^{6}$
${\;\;}^{48}$Sc	${\;\;}^{nat}$Ti	1.6 $\times \;10^{8}$	2.6 $\times \;10^{5}$	1.1 $\times \;10^{6}$
	${\;\;}^{nat}$V	2.5 $\times \;10^{8}$	-	1.7 $\times \;10^{6}$
	TiC	8.4 $\times \;10^{7}$	1.4 $\times \;10^{5}$	5.1 $\times \;10^{5}$
${\;\;}^{44}$Ti	${\;\;}^{nat}$Ti	1.5 $\times \;10^{4}$	1.9 $\times \;10^{1}$	2.5 $\times \;10^{2}$
	${\;\;}^{nat}$V	7.5 $\times \;10^{3}$	-	2.7 $\times \;10^{1}$
	TiC	7.5 $\times \;10^{3}$	1.3 $\times \;10^{1}$	1.2 $\times \;10^{2}$
${\;\;}^{47}$Ca	${\;\;}^{nat}$Ti	5.7 $\times 10^{6}$	7.5 $\times \;10^{-5}$	1.3 $\times \;10^{4}$
	${\;\;}^{nat}$V	5.8 $\times \;10^{6}$	-	7.7 $\times \;10^{3}$
	TiC	2.3 $\times \;10^{6}$	2.6 $\times \;10^{-2}$	6.1 $\times \;10^{3}$

**Table 5:** Target units with their configurations used in this study.

Target Unit Nr.	Ion Source	Configuration
685 M	Re surface	Standard. ${\;\;}^{45}$Sc sample.
686 M	W surface	Standard. ${\;\;}^{45}$Sc sample.
702 M	VADIS	Standard VD-5. ${\;\;}^{nat}$TiC target charge.
723 M	VADIS	Standard VD-5. ${\;\;}^{nat}$Ti-foil target charge.
741 M	VADIS	Standard VD-5. Gas-leak cooling structure with a thermocouple. ${\;\;}^{nat}$Ti-foil target charge.
766 M	VADIS	Standard VD-5. Gas-leak cooling structure. ${\;\;}^{nat}$V-foil target charge.
790 M	VADIS	W lining for VADIS cathode and transfer line. V foil lining inside target container. ${\;\;}^{nat}$V-foil target charge.
805 M	VADIS	Target container with transfer line heating from back. Standard ion source. Prototype heat screen assembly. ${\;\;}^{nat}$V-foil target charge.

**Table 6:** Target material and charge details for Sc production and release.

Material	Category	Melting Point, °C	Target Unit Nr.	Purity, %	Rolls or Pellets in Full Charge	Charge Mass, g	Charge Surface Area, m${\;\;}^{2}$	Charge Density ^1^, g/cm${\;\;}^{3}$
${\;\;}^{nat}$Ti	Metallic foils	1668	723M	99.6	12	74.8	1.11	1.19
			741M	99.6	12	96.3	1.43	1.53
${\;\;}^{nat}$V	Metallic foils	1910	766M	99.8 ^2^	11	44.8	0.59	0.71
			790M	99.8	10	61.7	0.81	0.98
			805M	>99.8	10	34.3	0.45	0.55
${\;\;}^{nat}$TiC	Carbides	3160	702M	99.5	69	29.2	72.4 ^3^	2.73

^1^ Rolled-foil charge density is considered as target material mass against the whole target-container volume; ${\;\;}^{nat}$TiC charge density is considered as ${\;\;}^{nat}$TiC pellet mass against internal-carbon-sleeve volume. ^2^ The foil surface was oxidized. ^3^ Measured and calculated for raw ${\;\;}^{nat}$TiC pill, outgassed at 300 °C for 19 h (2.48 m${\;\;}^{2}$g${\;\;}^{-1}$).

**Table 7:** Stable isotope and target material samples used for molecular beam studies.

Sample	Reactive or Buffer Gas	Observed Species of Interest	Temperature of Observation, °C
${\;\;}^{nat}$Ti foil	NF${\;\;}_{3}$, CF${\;\;}_{4}$	Ti${\;\;}^{+}$; TiF${\;\;}^{+}$; TIF${\;\;}_{2}^{+}$; TIF${\;\;}_{3}^{+}$; TiO${\;\;}^{+}$; TIO${\;\;}_{2}^{+}$; TIOF${\;\;}^{+}$; TIOF${\;\;}_{2}^{+}$	450
${\;\;}^{nat}$Ti foil	Cl${\;\;}_{2}$	Ti${\;\;}^{+}$; TiO${\;\;}^{+}$	1100
${\;\;}^{nat}$V foil	CF${\;\;}_{4}$	V${\;\;}^{+}$; VF${\;\;}^{+}$; VF${\;\;}_{2}^{+}$; VO${\;\;}^{+}$; VOF${\;\;}^{+}$	890
${\;\;}^{nat}$V foil	Cl${\;\;}_{2}$	V${\;\;}^{+}$; VCl${\;\;}^{+}$; VCl${\;\;}_{2}^{+}$; VO${\;\;}^{+}$; VO${\;\;}_{2}^{+}$; VOF${\;\;}^{+}$; CaCl${\;\;}^{+}$	1250 ^1^
${\;\;}^{nat}$TiC	CF${\;\;}_{4}$	Ti${\;\;}^{+}$; TiF${\;\;}^{+}$; TIF${\;\;}_{2}^{+}$; TIF${\;\;}_{3}^{+}$; TiO${\;\;}^{+}$; TIO${\;\;}_{2}^{+}$; TIOF${\;\;}^{+}$; TIOF${\;\;}_{2}^{+}$	730–780
Sc${\;\;}_{2}$O${\;\;}_{3}$ in 5% HNO${\;\;}_{3}$	NF${\;\;}_{3}$	Sc${\;\;}^{+}$; ScF${\;\;}^{+}$; ScF${\;\;}_{2}^{+}$	1340–1420
Sc${\;\;}_{2}$O${\;\;}_{3}$ in 5% HNO${\;\;}_{3}$	CF${\;\;}_{4}$	Sc${\;\;}^{+}$; ScF${\;\;}^{+}$; ScF${\;\;}_{2}^{+}$	1100–1160
ScF${\;\;}_{3}$	CF${\;\;}_{4}$, Ar	Sc${\;\;}^{+}$; ScF${\;\;}^{+}$; ScF${\;\;}_{2}^{+}$	350–400 ^2^
ScCl${\;\;}_{3}$ in 0.1M HCl	CF${\;\;}_{4}$, Ar	Not observed	-

^1^ Cl${\;\;}_{2}$ gas was first injected only at the indicated temperature. ^2^ Noticeable increase in beam intensity from pA to nA range was gained at 600–1000 °C.

## Data Availability

The raw data supporting the conclusions of this article will be made available by the authors on request.

## Abbreviations

The following abbreviations are used in this manuscript:

CERN	The European Organization for Nuclear Research
MEDICIS	Medical Isotopes Collected from ISOLDE
ISOLDE	Isotope Separation On-Line DEvice
MWCNT	Multi-Walled Carbon NanoTubes
ISOL	Isotope Separation On-Line
CNT	Carbon NanoTubes
CB	Carbon Black
CZT	Cadmium Zinc Telluride
MELISSA	MEDICIS Laser Ion Source Setup
IP	Ionization Potential
AIS	Auto-Ionization State
FEBIAD	Forced-Electron-Beam-Induced Arc Discharge
VADIS	Versatile-Arc-Discharge Ion Source
PSB	Proton Synchrotron Booster
HRS	High-Resolution Separator
GPS	General-Purpose Separator
ISIS	ISolde Irradiation Station
FLUKA	FLUktuierende KAskade
HPGe	High-Purity Germanium
MCNP	Monte Carlo N-Particle Transport
EOB	End Of Beam
TTY	Thick-Target Yield
